# Systematic Survey and Expression Analysis of the Glutaredoxin Gene Family in *Capsicum annuum* Under Hypoxia Stress

**DOI:** 10.3390/biology14091106

**Published:** 2025-08-22

**Authors:** Yixian Guo, Sirui Ma, Ziying Li, Yang Yu, Di Liu, Tianyi Zhang, Ruiwen Hu, Demian Zhou, Ying Zhou, Shi Xiao, Qinfang Chen, Lujun Yu

**Affiliations:** 1 State Key Laboratory of Biocontrol, Guangdong Provincial Key Laboratory of Plant Stress Biology, School of Life Sciences, Sun Yat-sen University, Guangzhou 510275, China; guoyx67@mail2.sysu.edu.cn (Y.G.); masr469@163.com (S.M.); lizy333@mail2.sysu.edu.cn (Z.L.); yangyuci@hotmail.com (Y.Y.); liud47@mail2.sysu.edu.cn (D.L.); skybruce091@sina.com (T.Z.); hrw807@163.com (R.H.); demychow@163.com (D.Z.); zhouying25@mail.sysu.edu.cn (Y.Z.); xiaoshi3@mail.sysu.edu.cn (S.X.); 2 School of Agriculture and Biotechnology, Shenzhen Campus of Sun Yat-sen University, Shenzhen 518107, China

**Keywords:** *GRX*, evolution, expression pattern, pepper, hypoxia

## Abstract

Glutaredoxins (GRXs) are important proteins in plant development and environmental adaptation. Despite extensive characterization of *GRX* gene family members in various plant species, limited research has been conducted on the identification and functional analysis of *GRXs* in the economically important Solanaceae family pepper (*Capsicum annuum*). This study identified 35 typical *GRX* genes in pepper and categorized them into three distinct groups: CC-, CGFS-, and CPYC-type, based on the phylogenetic topology, which was consistent with motif or domain arrangement, and gene structures. Furthermore, the determination of ω values indicated that purifying selection was a significant factor in the evolutionary diversification of *GRX* genes in the eudicot family. Intra-genome investigations demonstrated that both segmental and tandem duplications were involved in the expansion of *CaGRX* genes. Moreover, examination of collinearity within the Solanaceae family revealed 53 orthologous pairs of *GRX* genes. Additionally, prediction of *cis*-regulatory elements and analysis of expression profiles revealed the significant involvement of *GRX* genes in plant stress response, specifically in relation to hypoxia and submergence. Subsequent subcellular localization examination suggested *CaGRX* may be involved in the endomembrane system and regulation of oxidative balance in plants. Collectively, these findings enhance our comprehension of the structural and functional properties of *GRX* in pepper, and establish a groundwork for subsequent functional characterization of the *CaGRX* genes.

## Simple Summary

Glutaredoxins (GRXs) are essential for plant growth and environmental adaptation, yet their roles in pepper (*Capsicum annuum*) are not well understood. This study identified 35 *CaGRX* genes, classified into CC-, CGFS-, and CPYC-types based on phylogeny, conserved motifs, and gene structure. Evolutionary analysis showed strong purifying selection, with segmental and tandem duplications promoting gene expansion. Collinearity analysis revealed 53 orthologous *GRX* gene pairs across Solanaceae species. Cis-element prediction and expression profiling indicated that *CaGRX* genes respond to various stresses, particularly hypoxia and submergence. Subcellular localization suggested their involvement in the endomembrane system and oxidative stress regulation. These findings provide a basis for further functional studies of *GRX* genes in pepper.

## 1. Introduction

The accumulation of reactive oxygen species (ROS) in plants exposed to stress is a significant factor contributing to the reduction in global crop yields [1,2,3]. Glutaredoxins (GRXs) are involved in redox homeostasis and ROS signal transduction [4,5] by participating in the reversible reduction of disulfide bonds with the help of peptide reductase (GR) and glutathione (GSH), thus influencing NADPH and glutathione levels [6,7]. *GRX* genes mainly play a key role in scavenging active oxygen damage and are important antioxidant defense members in plants [1], which maintain and ensure the stability of redox homeostasis within plant cells [8,9].

The typical plant *GRX* gene family was categorized into three distinct groups, including CC-, CPYC-, and CGFS-type, based on phylogeny tree, domain organization, and active site motif composition of *GRX* [7,10,11]. CGFS- and CPYC-type *GRX* genes containing the CxxC/S and CGFS motifs were ubiquitously detected in all organisms, with CC-type *GRXs* sharing the CCxx motifs, only occurring in the plant [5,8,12,13,14]. It was reported that plants comprised many more *GRX* genes than mammals, bacteria, and fungi [7], because of gene expansion, especially CC-type *GRXs* in plant evolution [15].

The modulation of *GRX* genes in plant development and in response to stress exposure suggests the pivotal involvement of *GRXs* in these processes [8,16]. Nevertheless, limited research has been conducted on the biological functions and regulatory mechanisms of certain plant *GRX* genes in Arabidopsis and rice. *AtGRXS8* can impede the transcription and growth of primary nitrate responses by disrupting the activity of TGA1 and TGA4 [17]; In the Arabidopsis *atgrxs17* mutant, both cell proliferation and cell cycle are affected [18,19,20]; *ROXY1* and *ROXY2*, belonging to the CC-type *GRX* genes, jointly control the anther development of Arabidopsis, and the double mutants of *roxy1* and *roxy2* do not produce pollen and are sterile [20,21]; *GRXS13* protects *Arabidopsis thaliana* from damage caused by photooxidative stress, and contributes to the plant’s response to such stress [13]; TGA-interacting *GRX480/ROXY19* is involved in regulating the interaction between salicylic acid (SA) and jasmonic acid (JA) signal transduction pathway [22]. The overexpression of LOC_Os02g40500 and LOC_Os01g27140 has been shown to improve plant resistance to drought and heavy metal cadmium stresses [23,24]; the overexpression of *OsGRXC2.2* has been shown to impact the development, resulting in an increase in rice grain weight [25]; *OsGRX20* has been identified as a key regulator in improving rice resistance to various stresses, including bacterial blight, heavy metal toxicity, heat, and cold, thereby enhancing rice tolerance [26]; the interaction between *OsGRXS15* and *OsWRKY65* has been demonstrated to regulate *OsPR1*, ultimately enhancing plant disease resistance [27]; the overexpression of *OsGRX8* in Arabidopsis has been demonstrated to enhance resistance to stress, while RNAi rice plants have shown increased sensitivity to abiotic stresses [28]. *MALESTERILECONVERTEDANTHER1* (*MSCA1*), acting redundantly with two paralogues of *ZmGRX2* and *ZmGRX5*, regulates the redox balance during development and in response to stress [29]. *SlGRX1* plays a crucial role in the regulation of abiotic tolerance to combat oxidation, drought, and salt stresses [8,30]. The overexpression of chickpea *GRX* (LOC101493651) and *CsGRX4* in Arabidopsis demonstrate potential for enhancing plant resistance to heavy metals and regulating defense mechanisms against biotic stress, respectively [31,32]. *GRXS16* plays a crucial role in the metabolism of glutathione-dependent pesticides in tomatoes [33]; the overexpression of the pepper *CcGRXS12* gene can increase *Nicotiana benthamiana*’s resistance to plant viruses, H_2_O_2_, and herbicide paraquat [34].

The typical *GRX* gene family has been identified on a genome-wide scale in some plant species. There were 31 *GRX* genes identified in *Arabidopsis thaliana* [11], 41 *GRX* genes in *Populus trichocarpa* [35], 29 *GRX* genes in rice [7,12], 45 *GRX* genes in maize (*Zea mays*) [36], and 77 *GRX* genes in cotton (*Gossypium hirsutum*) [14], 38 *GRX* genes in banana (*Musa acuminata*) [37], 30 *GRX* genes in bean (*Phaseolus vulgaris*) [16], 39 *GRX* genes in cassava (*Manihot esculenta*) [38], 44 to 51 *GRX* genes identified in tomato (*Solanum lycopersicum*) and potato (*Solanum tuberosum*), respectively [8] Pepper (*Capsicum annuum*) is rich in various pharmacologically active compounds and an indispensable economic crop [39,40], whose development and yield were greatly influenced by abiotic stresses, especially flooding [41]. However, there was no *GRX* gene function or distribution information identified in pepper. In addition, the well-sequenced pepper genome data [42] offers the chance to investigate the *GRX* gene evolution events. A comprehensive analysis of the *CaGRX* genes in pepper was conducted to facilitate further research on their role.

Here, the comprehensive genome-wide *GRX* genes were detected in pepper, an important Solanaceae species, with domain organization, chromosome location, gene duplication detection, promoter analysis, protein subcellular localization experiment, and expression patterns. The findings will enhance understanding of the evolution of plant *GRX* genes and provide the *GRX* genes for subsequent functional investigation, upon the environmental stresses.

## 2. Materials and Methods

### 2.1. Identification of GRX Genes in Pepper, Conserved Domain Organization, and Promoter Analysis

Genome-wide search of the GRX proteins was conducted using the BLASTp program (NCBI). The Arabidopsis GRX genes were used as queries against the selected pepper (Capsicum annuum) “Zunla” genome of Solanaceae [43], with an E-value parameter of less than 10^−5^ [44]. PF00462 entry of the GRX domain in Pfam database (accessed on 12 December 2023) [45] was utilized as a seed to search GRX genes with HMMER 3.0 software [46], in conjunction with the Ensembl Plants (accessed on 12 December 2023) [47] and Phytozome databases (accessed on 12 December 2023) [48]. Subsequently, candidate intact GRX proteins were further screened using the SMART (accessed on 2 January 2024), CDD (accessed on 2 January 2024), and InterProscan (accessed on 2 January 2024) databases, as outlined previously [49,50]. The gene structure analysis was utilized the Gene Structure Display Server (GSDS2.0) to identify exon-intron organization. GRX domain organization was characterized by identifying conserved motifs through the MEME (multiple EM for motif elicitation) website (accessed on 01 February 2024) with classic mode and zoops (Zero or One Occurrence Per Sequence) as the site distribution [51]. Predictions of cis-regulatory elements (CRE) within the 2000 bp upstream of *GRX* gene promoters were made using the PlantCARE database (accessed on 3 February 2024) with default parameters [52]. The physical and chemical characteristics of CaGRX proteins were analyzed by the EXPASY website (accessed on 16 January 2024) [53]. The results were visualized using the TBtools software (version 2.331, South China Agricultural University, Guangzhou, China) [54].

### 2.2. GRX Gene Phylogenetic Tree and Duplication Analysis

The GRX domains were aligned using MUSCLE of the MEGA X software (version 11.0.13, Mega Limited, Auckland, New Zealand) [55], followed by phylogenetic tree construction, with the maximum likelihood method (ML), incorporating the Jones-Taylor-Thornton (JTT) model, Gamma distribution evolutionary rates (+G), pairwise deletions, and 1000 bootstraps [56,57]. The constructed tree was redrawn by FigTree (version 1.4.5, The University of Edinburgh, UK). Segmental and tandem duplication of *GRX* genes were further detected based on a previous study [44]. To identify syntenic blocks of the intra- and inter-genomic across the Solanaceae family, MCScanX (version 1.0.0, The University of Georgia, USA) was employed with the parameters as suggested [58].

### 2.3. GRXs Gene Expression Analysis in Pepper

The tissue-, phytohormone- or stress-specific expressions of pepper *GRX* genes were obtained from the published PepperHub database (http://www.hnivr.org/pepperhub, accessed on 10 May 2024) [43]. The RPKM value of *GRX* genes was further analyzed using a heatmap of the R software (version 4.1.3, R Foundation for Statistical Computing, Vienna, Austria), as outlined previously [59,60].

### 2.4. Submergence and Hypoxia Treatment and Quantitative RT-PCR

Pepper “Zhangshugang” was used for submergence and hypoxia treatment [61]. The pepper was grown under the conditions as outlined previously [62]. For the light submergence (LS) treatment, 4-week-old seedlings were immersed in water under 40 cm for durations of 12 h and 24 h, with samples collected from leaves, stems, and roots of 3 individual plants, as outlined previously [62]. The light hypoxia (LH) treatment entailed subjecting 4-week-old pepper plants to a hypoxia workstation with an oxygen concentration of 0.1% for durations of 12 h and 24 h, with samples collected from leaves, stems, and roots of 3 individual plants. The dynamic regulation of *CaGRX* genes under the LS and LH treatments was analyzed through qRT-PCR, following the methodology, as outlined previously [44,63]. Primers used are listed in Table S1.

### 2.5. CaGRX Subcellular Localization Analysis

To ascertain the localization of CaGRX15/24 in the cell, the complete coding sequence (CDS) was cloned using the specific primers (Table S1), and inserted into the pUC121-XTEN-GFP-HA vector. The resulting construct, designated as pUC121-CaGRX15/24-GFP-HA, facilitates the expression of a fusion protein consisting of CaGRX15/24-GFP. Arabidopsis protoplasts were then transfected with CaGRX15/24, which were imaged using a Zeiss LSM880 laser confocal microscope (Zeiss, Oberkochen, Germany) [64,65,66].

## 3. Results

### 3.1. Identification of GRX Members in Solanaceae Species Genomes

A thorough examination of the complete pepper (*Capsicum annuum*) genome of the *GRX* genes was conducted with BLASTP and HMMER 3.0 methods, followed by screening in CDD, SMART, and InterProscan databases, resulting in the identification of 35 *GRX* genes. The longest transcripts of these *GRX* genes were selected and subsequently designated based on genomic location (Table S2). Through the EXPASY website, the physical and chemical characteristics of CaGRX proteins were predicted, with a range of 100 to 219 amino acids and 11.0 to 24.5 kDa of molecular weight (MW). Notably, CaGRX06 and CaGRX31 exhibited substantial molecular weights of 47.9 kDa and 32.7 kDa, respectively. The isoelectric point (pI) was predicted to range from 5.0 to 9.5, with CaGRX31 being closest to 7 at 6.9 (Table S2).

### 3.2. Phylogenetic Relationship and Structure Analysis

**Figure 1:**
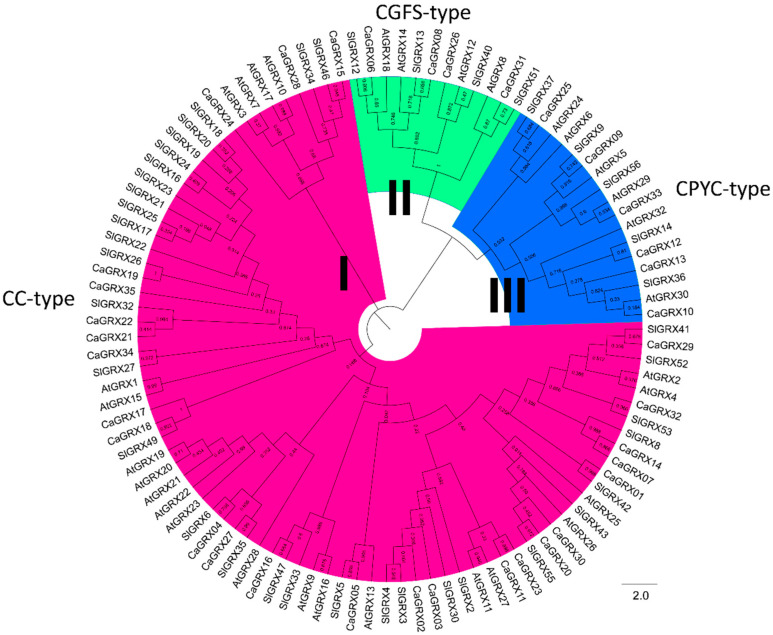
Phylogenetic Relationships among 107 Plant GRX Proteins. We assembled a phylogenetic tree of the GRX protein family in the selected genomes utilizing the MEGA X software suite, using the GRX catalytic domains. The GRX proteins were categorized into three discrete clusters, each denoted by a unique color. I, II, and III denote the CC-type, CGFS-type, and CYPC-type GRX genes, respectively. The phylogenetic tree was further refined for clarity and visual presentation using FigTree software.

To clarify the phylogenetic characteristics of plant *GRX* genes, MEGA X software was employed to draw the phylogenetic tree, using the catalytic domains of GRX proteins. Utilizing the Maximum Likelihood and JTT matrix-based model, the generated phylogenetic tree categorized the GRX proteins into three distinct groups labeled as Groups I–III. This classification was confirmed by the phylogenetic topology, as well as the organization of domains or motifs. Notably, the bootstrap values observed within each group were higher compared to those among groups (Figure 1), indicating the reasonableness of the classification.

**Figure 2:**
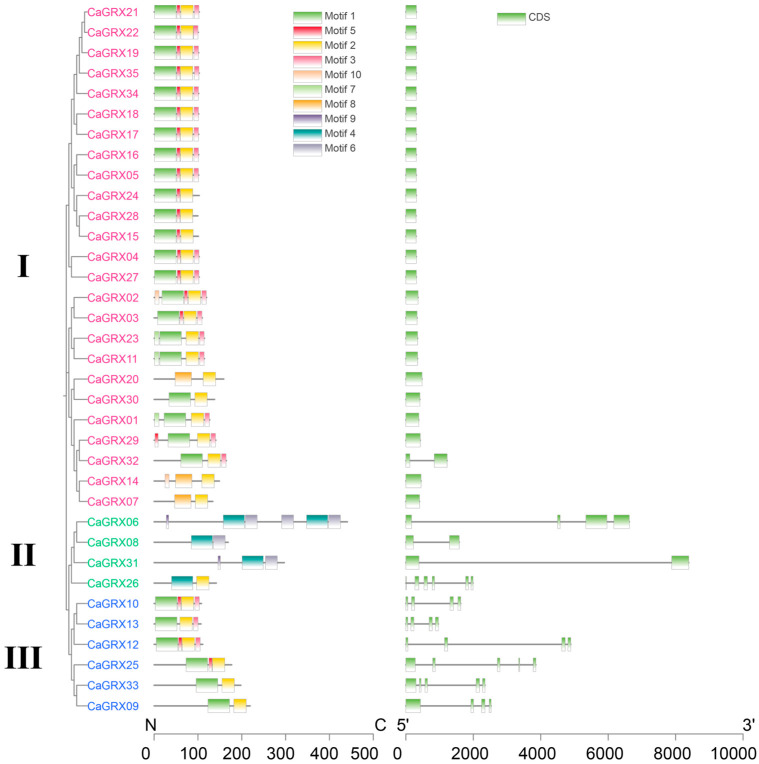
Comparative analysis of conserved motifs and gene architecture in pepper *GRX* family members. On the left, a subset phylogeny encompassing 35 *GRX* family members is reproduced from Figure 1 for reference. I, II, and III denote the CC-type, CGFS-type, and CYPC-type GRX genes, respectively. At the center, we display the conserved motif composition for the CaGRX proteins, as predicted by the MEME suite. Ten well-defined conserved motifs are illustrated using colored bars. The sequential arrangement of GRX proteins mirrors their phylogenetic relationships. On the right, the gene structure of *CaGRX* genes is depicted, providing insights into the exon-intron organization characteristic of this family.

Phylogenetic analysis revealed that typical GRX proteins were categorized into three groups, known as CC-, CGFS-, and CPYC-type, which was further supported by GRX protein domain organization and gene structure (Figure 2). By employing the MEME website, it was observed that there were higher E-values and conservation of motif organization among the groups compared to within groups (Figure 2), indicating a reliable classification. The GSDS website (accessed on 28 January 2024) was utilized for gene structure detection, revealing a higher degree of similarity within groups compared to between groups (Figure 2), consistent with the domain arrangement. The members of the *GRX* genes in pepper, tomato, and *Arabidopsis thaliana* were found across all three groups. Specifically, the *CaGRX* gene was present in 19, 4, and 6 genes within the three groups, *SlGRX* in 25, 4, and 5 genes within the three groups, and the *AtGRX* in 21, 4, and 6 genes within the three groups.

### 3.3. Pepper GRX Genes Location and Duplication

**Figure 3:**
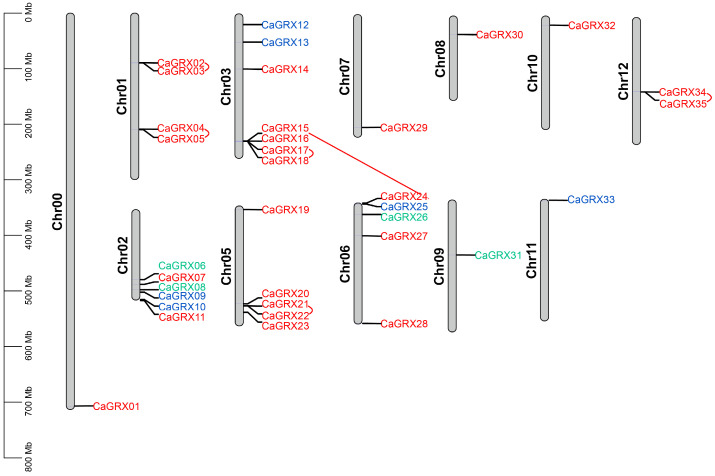
Chromosomal localization of pepper *GRX* genes. Chromosome numbers are displayed to the left of each chromosome schematic (vertical bars). Chromosomal dimensions correspond to their relative lengths, as determined from data in the Ensembl database. Tandemly duplicated and segmentally duplicated *GRX* gene pairs are denoted by connecting red arcs. The coloration of *GRX* gene markers reflects the three distinct *GRX* groups, consistent with the classification presented in Figure 1.

In order to ascertain the position and proliferation of pepper *GRX* genes, a comprehensive analysis was conducted wherein 35 *GRX* genes were assigned to their corresponding chromosomes, spanning a total of 12 chromosomes (Figure 3). The distribution of *CaGRX* genes on chromosomes exhibited non-uniformity, with 7 *CaGRX* genes situated on chromosome Chr03 and none on chromosome Chr04. Furthermore, in accordance with the criteria for duplications [49], a pair of segmental duplicate *CaGRX* genes was discerned between *CaGRX15* on chromosome Chr03 and *CaGRX24* on chromosome Chr06 (Figure 3). Additionally, five regions containing tandem duplicate *CaGRX* genes were detected on chromosomes 01, 03, 05, and 12 (Figure 3). The results indicated that segmental and tandem duplications had a substantial impact on the expansion of *GRX* genes in pepper, aligning with the evolutionary trends detected in other Solanaceae species.

The assessment of selective pressure on gene evolution entailed the computation of Ka, Ks, and ω (Ka/Ks ratio) values, as outlined previously [67,68]. According to the neutral theory proposed by Nei [69], the ω value serves as an indicator of selection. To evaluate the selection pressure of the *GRX* genes, pairwise comparisons were conducted among three groups as well as within species.

**Figure 4:**
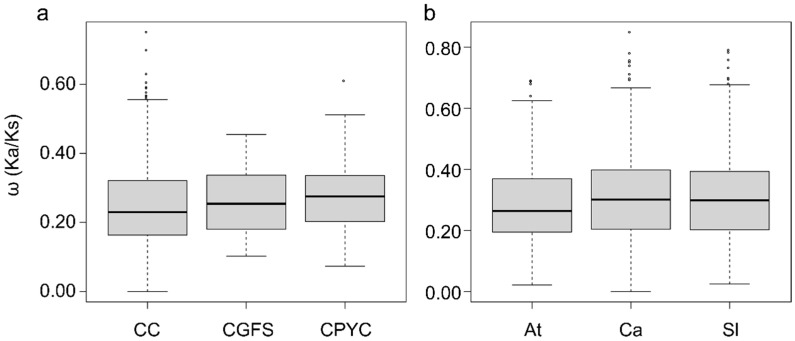
Illustration of the Ka/Ks ratios (ω) of *GRX* genes. The distribution of nonsynonymous to synonymous substitution ratios (Ka/Ks), or ω values, for *GRX* genes in groups and plant genomes is presented. (**a**) The Ka/Ks values were derived from pairwise comparisons among members of groups of CC-type, CGFS-type, and CPYC-type. (**b**) Ka/Ks distributions are shown for pairwise comparisons within the genomes of Arabidopsis (*At*), pepper (*Ca*), and tomato (*Sl*). The *Y*-axis denotes the Ka/Ks ratios for each gene pair, with box plots generated using the R statistical program.

The findings demonstrated that the ω values for the analyzed *GRX* gene pairs consistently fall below 1, indicating a pattern of purifying selection. Furthermore, the Ka/Ks ratio remained below 1 across the three groups, indicating varying levels of purifying selection pressures, with none of positive selection observed, within the Solanaceae family. Additionally, the average values of CC-, CGFS-, and CPYC-type *GRX* genes were determined to be 0.25, 0.26, and 0.28, respectively (Figure 4a). These findings indicate a consistent level of stringent purification selection across the three groups. Additionally, the average values of *At*, *Ca*, and *Sl GRX* genes were calculated as 0.28, 0.31, and 0.31, respectively (Figure 4b), with comparable Ka/Ks ratios all below 1. This suggests a high likelihood of functional divergence during the evolutionary process of the *GRX* genes.

### 3.4. Collinearity Analysis of Solanaceae GRX Genes

**Figure 5:**
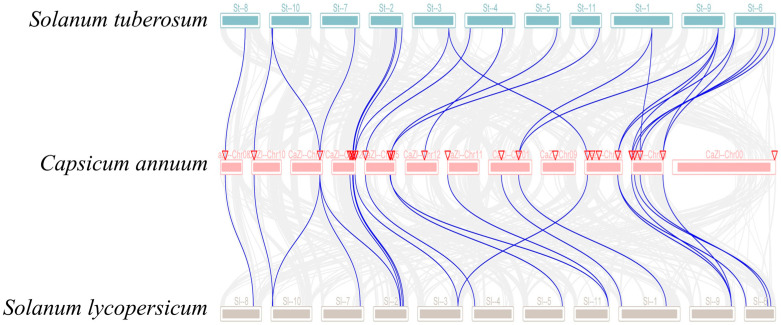
Collinearity analysis of *GRX* genes between pepper, tomato, and potato. Chromosomes from three distinct Solanaceae species are depicted as colored boxes: *Solanum tuberosum* (potato) chromosomes are shown in blue at the top, while *Capsicum annuum* (pepper) chromosomes are represented in pink at the middle, and *Solanum lycopersicum* (tomato) chromosomes are shown in brown at the bottom. Putative orthologous *GRX* genes across the genomes are connected with lines, as identified by the MCScanX software. The innermost grey solid lines highlight synteny among the *GRX* genes. In total, 24 orthologous *GRX* gene pairs were identified between tomato and pepper, and 29 pairs between pepper and potato, denoted by blue solid lines indicating their orthologous relationships.

In order to further investigate the evolutionary relationships among Solanaceae family species, an analysis of interspecific *GRX* gene collinearity was conducted utilizing the MCScanX software. Totally, 53 collinear *GRX* genes were detected across pepper, tomato, and potato, with e-values below 1e^−10^ (Figure 5) [62]. Specifically, 24 collinear genes were observed between pepper and potato, and 29 collinear genes were detected between pepper and tomato (Figure 5). The presence of numerous homologous *GRX* gene pairs within the *GRX* gene family in Solanaceae indicates a substantial degree of conservation.

### 3.5. CRE Analysis of the CaGRX Gene Promoters

**Figure 6:**
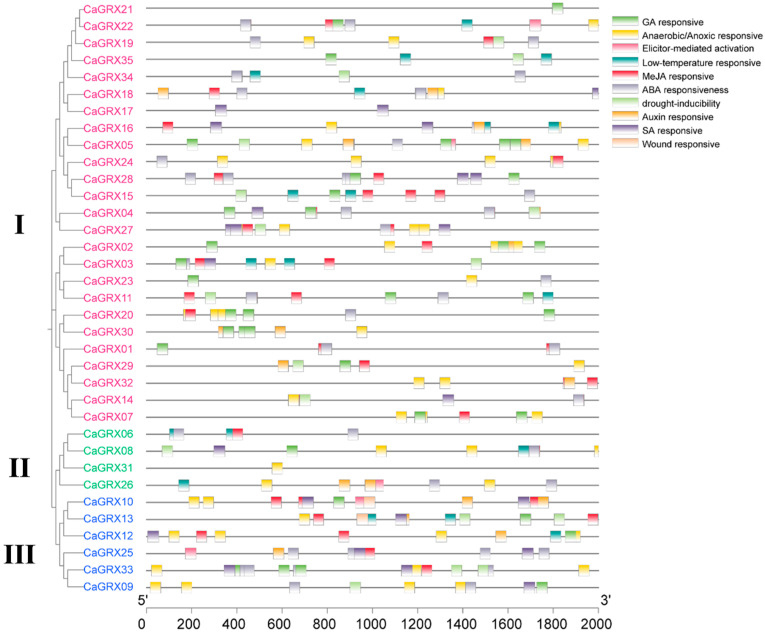
Prediction and analysis of *cis*-regulatory elements (CREs) located within the promoter sequences of the *GRX* genes. On the left side, a phylogenetic tree of the pepper *GRX* family isadapted from Figure 1. I, II, and III denote the CC-type, CGFS-type, and CYPC-type GRX genes, respectively. On the right side, the PlantCare database was employed to forecast the CREs present in the 2000 bp upstream regions of the 35 *CaGRX* genes. These CREs could be classified into two primary groups: phytohormone-related elements (such as ABA, Auxin, GA, MeJA, and SA), and stress-related elements (such as drought, low-temperature, elicitor-mediated activation, and wound).

To enhance comprehension of *GRX* gene roles in pepper response to stresses, an examination of the 2 kb promoter of the *CaGRX* gene was undertaken utilizing the PlantCARE. The analysis unveiled the existence of 322 CREs within the promoter regions of 35 *CaGRX* genes that exhibited responsiveness to plant hormones and stress. Specially, a total of 53 Anaerobic/Anoxic responsive-related cis-regulatory elements (CREs), 5 Elicitor-mediated activation CREs, 20 Low-temperature responsive-related CREs, 96 MeJA responsive-related CREs, 2 Wound responsive-related CREs, 51 ABA responsive-related CREs, 18 Auxin responsive-related CREs, 37 GA responsive-related CREs, 23 SA responsive-related CREs, and 17 drought inducibility-related CREs were identified in the *CaGRX* gene (Figure 6). Additionally, the majority of *CaGRX* genes contain multiple CREs responsive to hormones or stress, with the exceptions of CGFS-type *CaGRX31* containing only one Anaerobic/Anoxic-responsive-related CRE and CC-type *CaGRX21* containing only one GA responsive-related CRE (Figure 6).

The study revealed significant variations in the abundance and diversity of cis-acting elements among CC-type, CPYC-type, and CGFS-type. Specifically, 214 CREs were identified in CC-type, with the highest number of Anaerobic/Anoxic responsive elements found in abiotic stress (33 genes) and MeJA responsive elements in hormones (68 genes) (Figure 6). In the CPYC-type, 80 cis-acting elements were detected, with the highest number of Anaerobic/Anoxic responsive elements in abiotic stress (14 genes) and MeJA responsive elements in hormones (22 genes) (Figure 6).

Totally, 28 CREs were detected in CGFS-type *CaGRX* genes, with the highest number of Anaerobic/Anoxic responsive-related elements observed in abiotic stress (6 genes), and MeJA and ABA responsive elements found in hormone regulation (6 genes) (Figure 6). In contrast, CC-type *CaGRX* genes exhibited the highest abundance of cis-acting elements, with wound-responsive-related elements present exclusively in CPYC-type *CaGRX10* and *CaGRX13*, but not in the other two groups (Figure 6). These findings suggest that *CaGRX* genes within the Solanaceae family may be involved in various stress response pathways.

### 3.6. Expression Profile Analysis of CaGRX Genes

**Figure 7:**
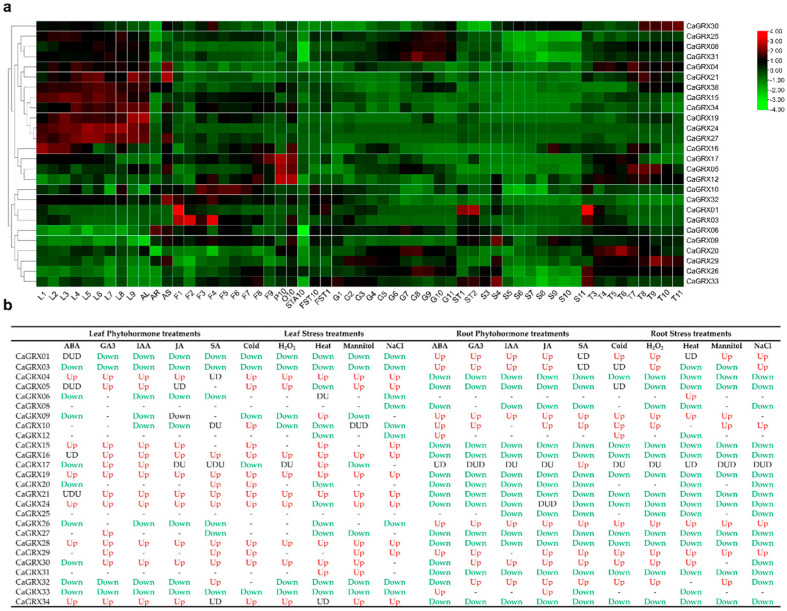
Expression profile of 26 *CaGRX* genes in different plant developmental stages and in response to phytohormone and stress treatments. (**a**) Expression profiles of 26 *CaGRXs* in plant developmental stages obtained from the Pepperhub database. L: leaf; F: flower; P: Petal; O: Ovary; STA: Anther; FST: Whole Fruit; G: Pericarp; T: Placenta; ST: Placenta and Seed; S: Seed. Heatmap clustering was employed to visualize the expression values, with the color bar indicating the Z score of the expression value. (**b**) The regulatory effects of phytohormone and stress treatments on *CaGRXs* genes in leaves (L) and roots (R), which included ABA, GA3, IAA, JA, SA, and cold, H_2_O_2_, Heat, mannitol, and NaCl. Up (Red) and Down (Green) indicated the up-regulated and down-regulated genes, with “-“ showing no regulation.

In order to investigate the function of *CaGRX* genes in development, expression analysis from the PepperHub database was conducted [43], as previously published articles [62]. Our findings revealed that *CaGRX14* exhibited low expression levels in seeds (S3, S4, S5), while *CaGRX22* was specifically expressed in leaves. Additionally, *CaGRX18*/23/*02/07/11/13/35* genes showed either no expression or RPKM values below 1.0. The expression patterns of 26 *CaGRX* genes across 57 tissues were clustered and visualized using heatmaps. The Tissue specificity index (TAU) indicated varying levels of expression priority among different groups. Furthermore, the expression of *CaGRX* was found to be highest in reproductive tissues across all three types (Figure 7a). The *CaGRX* genes within the same group show varying expression patterns, indicating potential functional diversification or sub-functionalization. Specifically, in the CC-type, the majority of CC-type *CaGRX* genes showed primary expression in leaves, with *CaGRX02/03* in flowers, and *CaGRX05/17* in petals. Within the CGFS-type, *CaGRX08/31* were predominantly expressed in pericarp, *CaGRX06* in AR, and *CaGRX26* in Placenta (P). In the CPYC-type, *CaGRX09/13/33* exhibited preferential expression in seeds, *CaGRX10* in flowers, *CaGRX12* in petals (P10), and *CaGRX25* in pericarp (G10) (Figure 7a).

### 3.7. Expression Profiles of CaGRX Genes Under Phytohormone and Stress

Previous experimental studies have highlighted the significant involvement of *GRX* genes in plant response to stress [70]. To further explore the roles of *CaGRX* genes, expression data in the PepperHub were retrieved, which encompasses information on pepper exposed to different phytohormones and stress [43]. The findings indicated that the majority of *CaGRX* genes exhibited induction by treatments, with the exception of *CaGRX08/18/25* (Figure 7b).

The expression patterns of *CaGRX* genes were found to be modulated by a variety of phytohormones both in roots and leaves. Specifically, *CaGRX01/03/09/10/26* were induced by ABA, GA, IAA, JA, and SA in the root. Furthermore, *CaGRX12* was up-regulated by ABA, with *CaGRX24* induced by JA in the root. Conversely, a significant proportion of *GRX* genes in pepper leaves were induced without repression by phytohormones, demonstrating a regulatory pattern distinct from the root. Notably, *CaGRX32* exhibited induction by SA, *CaGRX01* by ABA, and *CaGRX04/16/19/21/24/28* by all five phytohormones in leaves.

In root tissues, the expression levels of *CaGRX04/15/16/19/21/24/27/28/31/34* were significantly decreased in response to freezing, H_2_O_2_, salt, mannitol, and heat stress, while *CaGRX01* showed consistent up-regulation under these stress conditions. *CaGRX09* exhibited up-regulation by stress, except for NaCl. *CaGRX06* was specifically regulated by heat in both roots and leaves. *CaGRX04/16/19/21/28/34* were induced by all stress treatments in leaves, while *CaGRX01/03/33* were consistently suppressed.

*CaGRX09* was up-regulated under heat, but down-regulated under cold in leaves. Location on chromosome Chr01, *CaGRX02,* and *CaGRX03* formed a tandem duplicate gene pair, with *CaGRX02* showing lower expression levels in tissues compared to *CaGRX03* in F1, F3, and F4 (Figure 7a), indicating functional differentiation. The expression of *CaGRX03* in roots is up-regulated following hormone and stress treatments, except in response to heat and mannitol treatments (Figure 7). On chromosome Chr02, the tandem duplicate gene pair, *CaGRX04* and *CaGRX05,* displayed consistent expression patterns across various treatments, except for divergent expression patterns observed under heat stress in leaves and cold stress in roots. Meanwhile, on chromosome Chr03, the tandem duplicate genes *CaGRX17* and *CaGRX18* were identified. The expression of *CaGRX17* was upregulated in either roots or leaves following treatment, whereas *CaGRX18* remained unexpressed. *CaGRX21* and *CaGRX22* are located in tandem repeat on chromosome Chr04, with *CaGRX21* exhibiting increased expression in response to hormone treatment or stress, while *CaGRX22* remains unexpressed. Additionally, *CaGRX34* and *CaGRX35* are tandem duplicate genes on chromosome Chr07, with *CaGRX35* showing low expression in tissues, while *CaGRX34* displayed increased expression in leaves following hormone or stress treatment, but decreased expression in roots. In contrast, *CaGRX15* and *CaGRX24* were the only pair of segmental duplicate genes on pepper chromosomes, exhibiting comparable expression patterns. It is notable that the expression of the majority of genes increased in stems and decreased in roots following hormone treatment or stress. The distinct expression profiles of *CaGRX* genes in roots and leaves, exposed to phytohormones and stresses, suggested the presence of tissue-specific modulation strategies.

### 3.8. Pepper GRX Genes in Response to Hypoxia and Submergence

**Figure 8:**
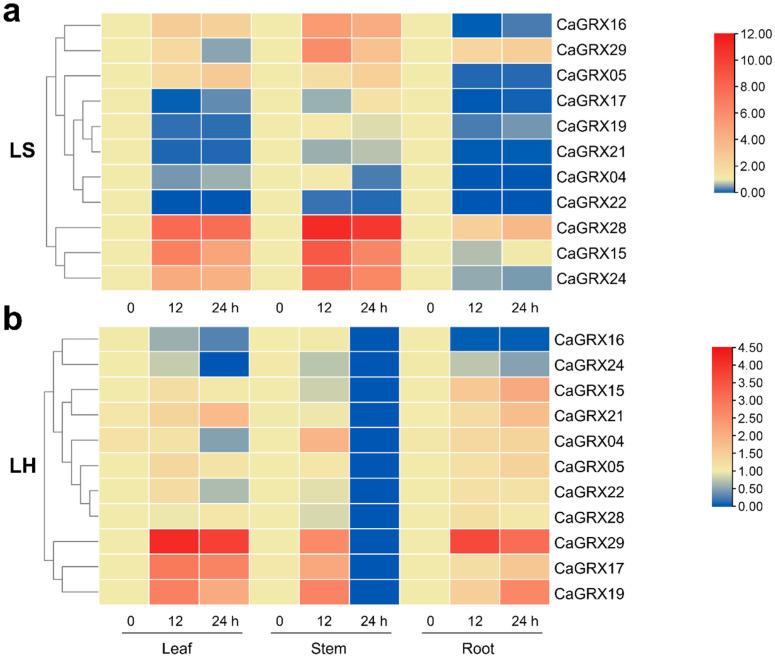
Expression patterns of *CaGRX* genes in response to submergence and hypoxia treatments. We conducted qRT-PCR analyses to investigate the expression dynamics of 11 *CaGRX* genes following 12 and 24 h of submergence and hypoxia treatments. (**a**) The relative expression levels of *CaGRX* genes in pepper leaf, stem, and root subjected to 12 and 24 h of submergence. (**b**) The relative expression levels of *CaGRX* genes in pepper leaf, stem, and root subjected to 12 and 24 h of hypoxia stress. The plants were grown under controlled greenhouse conditions with a diurnal temperature cycle of 28/23 °C (light/dark) and a photoperiod of 10 h of light followed by 14 h of darkness. Expression levels were normalized to the *CaUBI* gene as an internal control. The data points are presented as the mean of three biological replicates.

To further examine the effects of submergence and hypoxia stresses on the expression of *CaGRX* genes, four-week-old pepper plants were subjected to light submergence (LS) and light hypoxia (LH) treatments lasting 12 h and 24 h, respectively. The qRT-PCR was employed to detect the regulatory profiles of *CaGRX* genes, with the outcomes presented in a heatmap format (Figure 8a,b and Figure S1).

After treatment, the transcription levels of *CaGRX29* in leaf tissues were found to be increased in response to both LS and LH stress. Additionally, the transcription levels of *CaGRX05*, *CaGRX15*, and *CaGRX28* were specifically up-regulated following LS treatment, with no significant changes observed following LH treatment. The transcription levels of *CaGRX16* and *CaGRX24* were induced by LS treatment but repressed by LH treatment. In contrast, the expression of *CaGRX17*, *CaGRX19,* and *CaGRX21* decreased after LS treatment and up-regulated after LH treatment (Figure 8a,b and Figure S1).

In the stem, the transcription levels of various *CaGRX* genes were found to be modulated in response to treatments with LS and LH. Specifically, *CaGRX29* exhibited up-regulation following both LS and LH treatment. *CaGRX05*, *CaGRX15*, *CaGRX16*, *CaGRX24,* and *CaGRX28* displayed up-regulation after LS treatment, with no significant changes after LH treatment. In contrast, *CaGRX17* and *CaGRX19* did not show significant alterations following LS treatment, but were induced by LH treatment. *CaGRX04* was repressed by LS treatment, but repressed by LH treatment (Figure 8a,b and Figure S1).

In the root, the transcription levels of *CaGRX29* were up-regulated following both LS and LH treatments. *CaGRX28* exhibited up-regulation after LS treatment, with no significant changes observed after LH treatment. *CaGRX15* displayed no significant alteration following LS treatment, but showed up-regulation after LH treatment. Conversely, *CaGRX17*, *CaGRX19*, and *CaGRX21* were down-regulated after LS treatment and up-regulated after LH treatment (Figure 8a,b and Figure S1).

The expression profiles of CC-type members exhibited notable variations under LS and LH treatments at various time points, indicating a diverse array of expression patterns. Specifically, the up-regulation of *CaGRX29* at the transcriptional level in roots, stems, and leaves significantly increased following exposure to LS and LH treatments (Figure 8a,b and Figure S1), indicating a potential role in pepper exposed to hypoxia and water flooding stress. These findings suggest that the activation of *CaGRX* genes may contribute to the plant’s adaptive mechanisms in coping with hypoxia and subsequent recovery.

Gene duplication could lead to either redundancy or diversification of gene functions [12]. The segmental duplicate gene pair *CaGRX15/24* exhibited a comparable expression pattern during submergence stress (Figure 8a,b and Figure S1), but displayed temporal alternation under hypoxic stress. The fluctuation in expression levels of these genes was observed to be dependent on the duration of treatment. Particularly, in the context of submergence stress, the expression levels of *CaGRX15/24/28/29* showed a significant increase (Figure 8a,b and Figure S1). The overexpression of these genes may potentially contribute to aiding plants maintain redox homeostasis under stress.

### 3.9. Subcellular Localization of Selected CaGRX

**Figure 9:**
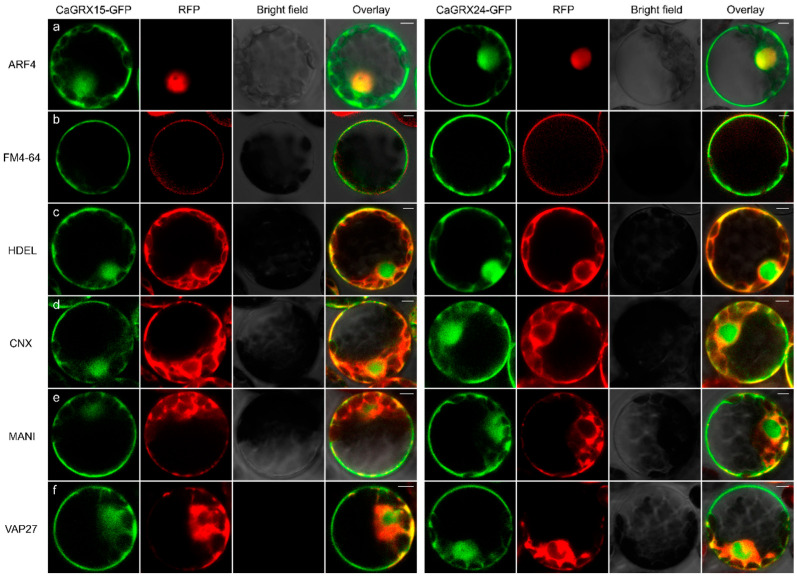
Subcellular localization of CaGRX15/24. (**a**) The expression of CaGRX15/24-GFP and the nucleus marker ARF4-mCherry was observed, along with their merged images. (**b**) The expression of CaGRX15/24-GFP and dye FM4-64 was observed, along with their merged images. (**c**) The expression of CaGRX15/24-GFP and dye Endoplasmic reticulum marker HDEL-mCherry was observed, along with their merged images. (**d**) The expression of CaGRX15/24-GFP and dye Endoplasmic reticulum marker CNX-mCherry were observed, along with their merged images. (**e**) The expression of CaGRX15/24-GFP and Golgi apparatus marker MANI-mCherry was observed, along with their merged images. (**f**) The expression of CaGRX15/24-GFP and ER-PM Contact Sites marker VAP27-mCherry was observed, along with their merged images. The fluorescence signal of CaGRX15/24 was predominantly localized in the nucleus and plasma membrane. Scale bars, 5 μm.

The localization of the CaGRX proteins in the cell was examined using the laser confocal microscope. To further analyze CaGRX15 and CaGRX24 proteins localization, the segmental duplication gene pair, fusion proteins with GFP tag were expressed heterologously in Arabidopsis protoplasts. The GFP fusion proteins co-localized with the markers could be identified by the channel merging, depicted in yellow color. The fluorescence emitted by CaGRX15/24-GFP was observed in the cytoplasm and cell membrane, co-localizing with a nuclear marker and a cytoplasmic membrane dye, FM4-64 (Figure 9a,b). It is established that HDEL is situated in the endoplasmic reticulum [71], while the calcium junction protein (CNX) serves as a lectin chaperone protein within the endoplasmic reticulum [72], facilitating peptide folding in the endoplasmic reticulum. The subcellular localization of the endoplasmic reticulum can be observed using fluorescent markers such as HDEL-RFP and CNX-RFP. The fluorescence distribution pattern of CaGRX15/24-GFP demonstrates a network-like arrangement within the endoplasmic reticulum (ER), colocalizing with HDEL-RFP and CNX-RFP (Figure 9c,d). Additionally, CaGRX15/24-GFP showed co-localization with the Golgi marker ManI and VAP27 (Figure 9e,f), a protein found on punctate structures linking the endoplasmic reticulum and the plasma membrane (ER-PM), in close proximity to microtubules and microfilaments [3]. The findings indicated that CaGRX15 and CaGRX24 present a similar subcellular location, suggesting the functional redundancy between the segmental duplication *CaGRX* pair.

## 4. Discussion

*GRX* genes have been experimentally demonstrated to be crucial for development and for combating stresses [8,16]. To date, genome-level identification has revealed 31 typical *GRX* genes in *Arabidopsis thaliana* [11], 41 genes in *Populus trichocarpa* [35], 29 genes in rice [7,12], 45 genes in maize [36], 77 genes in cotton [14], 38 genes in banana (*Musa acuminata*) [37], 30 genes in bean (*Phaseolus vulgaris*) [16], and 39 genes in cassava (*Manihot esculenta*) [38]. Additionally, researchers have identified *GRX* genes in the Solanaceae family species, including tomato and potato [8]. Despite extensive research on the genome of chili peppers [73], the precise count of the *GRX* genes in this species remains unknown.

This study aims to uncover the *GRX* genes in pepper, analyze their evolutionary traits, and investigate their functions in development and combating stresses. There were 35 *GRX* genes discovered in pepper, with a particular emphasis on their evolutionary characteristics, stress response mechanisms, and subcellular localization in protoplasts. This study holds significance in elucidating the molecular process involved in pepper plants’ adaptation to environmental stress, maintenance of redox balance, and regulation development.

### 4.1. The CaGRX Genes Expansion Characterization

There were 35 *GRX* genes identified within the pepper genome, falling into three groups based on the phylogenetic relationship, including Group I (CC-type), Group II (CGFS-type), and Group III (CPYC-type) (Figure 1). This classification aligns perfectly with the organization of gene structures, domains, or motifs (Figure 1 and Figure 2). The grouping of pepper *GRX* genes was in accordance with previous classifications of monocotyledon and dicotyledon *GRX* genes [8,11,12,14,16,36,38], demonstrating a similar grouping pattern observed in seed plants. Notably, the analysis revealed many more CC-type genes than the other two groups, in both monocotyledonous and dicotyledonous plants (Figure 1), suggesting diverse functions for this group.

Evaluation of selection pressure on the *GRX* gene within the groups and between species indicated ω consistently less than 1 (Figure 4), suggesting purifying selection on the Solanaceae *GRX* gene during evolution. Furthermore, the analysis revealed that the CC-type exhibited a lower ratio compared to the other two groups, indicating that this group underwent a more intense purifying selection than the CGFS- and CPYC-type.

The ω values were utilized as parameters to assess the selective pressure impacting gene evolution [49,67,68]. The results demonstrated that the ω values of *GRX* gene pairs within the CC-, CGFS-, and CPYC- types, as well as across the species Arabidopsis, pepper, and tomato, were all below 1.0 (Figure 4), indicating that *GRX* genes in Solanaceae underwent purifying selection during gene expansion, consistent with Arabidopsis. The Ka/Ks ratios among the three groups of *GRX* genes were comparable (Figure 4), indicating a similar level of purifying selection upon them. Furthermore, the ω values of *GRX* genes in pepper and tomato were observed to be elevated in comparison to those in Arabidopsis, suggesting that *SolGRX* genes have undergone a relatively reduced intensity of purifying selection.

### 4.2. Solanaceae GRX Duplications and Collinearity Analysis

Within the pepper genome, 35 *GRX* genes were characterized, exhibiting an uneven chromosomal distribution, which was also found in the tomato genome [8]. Nonetheless, this distribution pattern diverged from those expected in the context of diploidization or polyploidization events [74,75,76]. Five tandem duplication and one segmental duplication *CaGRX* gene pairs were identified (Figure 3), whose pattern was consistent with that found in tomato [8]. The pepper tandem and segmental duplication genes all belong to the CC-type, in line with the findings in tomato [8], suggesting their contribution to the proliferation of CC-type genes in the Solanaceae family species. Examination of the ω values for all pepper *GRX* gene pairs revealed that they were below the threshold of 1.0 (Figure 4), indicating negative selection. Interestingly, one CC-type *GRX* pair in tomato showed a ω value exceeding 1.0, indicative of positive selection, suggesting a subtle divergence in the evolutionary trajectory of *GRX* genes between pepper and tomato.

The collinearity analysis performed within the Solanaceae family has revealed that paralogous *GRX* genes share a striking resemblance in their gene structures and domain organization, predominantly among those genes classified in the same groups. This observed similarity was further corroborated by significantly elevated bootstrap values in the phylogenetic tree (Figure 1, Figure 2 and Figure 5). Overall, the results suggested that there have been no significant instances of motif or domain acquisitions or losses throughout the evolutionary history of the *GRX* genes.

### 4.3. CaGRX Genes Roles in Plant Response to Stress

Currently, numerous studies have revealed the involvement of the GRX protein in plant development and its adaptive response to stress [8,16]. The role of several CC-type *GRX* genes has been elucidated in Arabidopsis. The heterologous overexpression of *OsROXY1*, *OsROXY2,* and *ROXY1* in *Arabidopsis thaliana* resulted in heightened susceptibility to *Botrytis cinerea* [77]. *ATGRXS13* mutant plants exhibited resistance to *Botrytis cinerea* and were involved in ameliorating photooxidative stress [13,78]. In *atgrxs17* mutants, cell proliferation and the cell cycle were impacted [18,19,20]. The activity of *AtGRXS8* could inhibit primary nitrate response and subsequently influence root growth [17]. The transcription of *GRX480* could be induced by salicylic acid (SA) and counteract the jasmonic acid (JA) [22].

Elevated expression levels of *GRX* have been demonstrated to bolster plant resilience against stress. The overexpression of genes *LOC_Os02g40500* and *LOC_Os01g27140* has been found to significantly enhance plant tolerance to both drought and cadmium stress [23,24]. The CPYC-type *GRX* gene *OsGRX20* exhibited resilience to bacterial blight, heavy metal toxicity, as well as heat and cold stress [26]. Transgenic rice containing the CGFS-type *GRX* gene *OsGRXS15* demonstrates resistance to *Xanthomonas oryzae pv.oryzae* (*Xoo*) and *Fusarium fujikuroi* [27]. Furthermore, transgenic *Arabidopsis thaliana* plants overexpressing the CC-type *GRX* gene *OsGRX8* exhibited heightened resistance to salt, osmotic stress, and oxidative stress [28]. Elevated expression level of *OsGRXC2.2*, belonging to the CPYC-type, could increase rice grain weight [25].

The CC-type *GRX* gene *ZmGRX2*, *ZmGRX5,* and *MSCA1* were involved in regulating corn ear development and participated in redox reaction [29]. The suppression of the CC-type *GRX* gene *SlGRXC6* has been observed to result in heightened susceptibility to *tomato yellow leaf curl virus* (TYLCV) [79]. Recent studies have revealed that the elevation of the CGFS-type gene *CcGRXS12* could suppress the accumulation of *Pepper mild mottle virus* (PMMoV-I) [34]. Additionally, the tomato CC-type *GRX* genes *SlGRX21* and *SlGRX2* could be induced by *LncRNA16397*, resulting in the reduction of ROS and elevated resistance to phytophthora [80].

To date, the roles of *GRX* during hypoxia response are still unclear. The involvement of *CaGRX* genes exposed to both stresses could be assessed through expression data in the PepperHub database [43], as used in a previously published study [62]. An analysis of expression profile has revealed that the majority of *CaGRX* genes were predominantly active in reproductive tissues, including flower, petal, pericarp, placenta, and seed. This pattern suggested a similar involvement of *CaGRX* gene processes, such as flowering and seed ripening, aligning with known functions of *GRX* genes in banana [37]. Through a comprehensive analysis of CREs in the promoter, online expression profiles, and gene expression patterns following stress treatment, a novel interpretation of the role of the pepper *GRX* gene under stress conditions is presented.

Insight into the function of the *GRX* gene in peppers has the potential to inform breeding efforts aimed at developing stress-resistant varieties and enhancing crop yield and quality. Analysis of online expression profile data revealed distinct responses of different *CaGRX* gene groups to plant hormones and stress stimuli. Following hypoxia and flooding treatments, it was observed that 11 CC-type *GRX* genes within the same group exhibited varying responses to abiotic stress, leading to different expression levels. Subsequent amplification of these genes resulted in functional differentiation or sub-functionalization.

*GRX* is an oxidoreductase enzyme that exhibits catalytic activity. The determination of its subcellular localization within cells is crucial for accurately identifying its putative target protein and enhancing the analysis of its catalytic function. Confocal imaging results showed that CaGRX15 and CaGRX24 are predominantly nuclear and plasma membrane (Figure 9). In addition, a fraction of the protein is detected on membranes of the ER and Golgi apparatus, as well as at ER-PM contact sites (Figure 9). Experimental findings indicated that CaGRX15/24 was co-localized with the HDEL and CNX, which are specifically localized to the endoplasmic reticulum (ER) (Figure 9). The ER’s dynamic and intricate network structure is integral to the way plant cells respond to environmental stress, representing a unique endomembrane system, which is likely to contribute significantly to the defense mechanisms of higher plants [81,82]. The localization of the VAP27 protein at the interface of the VAP27 protein is located at the junction of endoplasmic reticulum and plasma membrane in *Arabidopsis thaliana* has been shown to impact root hair development [83]. VAP27 protein contributes to regulating Exo84c turnover through autophagy, ultimately influencing plant pollination duration and seed production [84]. It is proposed that maintaining the stability of VAP27 is crucial for proper plant development. Additionally, *GRXC1* was partially tethered to the membrane through its N-terminal myristoyl group and is localized to the ER [85]; GRXC4 has been identified as a substrate for vacuole sorting [86], while GRXC3/4 is anchored to the ER membrane, with its N-terminal domain displaying catalytic activity within the early stage of the secretory pathway [85]. Previous research has pinpointed the presence of both HDEL and VAP27 at the ER-plasma membrane interface [84,87]. It is suggested that CaGRX may engage with yet unidentified proteins to constitute a pre-existing platform, which contributes to oxidative stress response or catalyzes crucial processes. The findings suggested that *CaGRX* may have a significant influence on plant development and its capacity to adapt to stress.

## 5. Conclusions

This study firstly characterized 35 typical *GRX* genes in the pepper genome, which were categorized into three distinct groups, based on the phylogenetic tree topology, aligning with the organization of gene structures, domains, or motifs. Furthermore, the calculation of ω values had shed light on the predominant role of purifying selection in the evolutionary expansion of the GRX gene family. Further intra-genome analysis further illustrated that the significant contributions of both tandem and segmental duplications to the proliferation of *CaGRX* genes, with 53 orthologous pairs of *GRX* genes identified using collinearity analysis. Additionally, assessment of online expression profiles and *CaGRX* expression levels following submergence and hypoxia stress revealed the significant involvement of *GRX* genes in plant response to stress, particularly in the context of hypoxia induced by environmental factors. By examining subcellular localization and co-localization of *CaGRX* with different membrane markers, it was hypothesized that *CaGRX* may contribute to the endomembrane system and regulate oxidative equilibrium in plants. These discoveries enhanced our comprehension of the structural and functional aspects of *GRX* in pepper, and established a groundwork for subsequent functional characterization of the *CaGRX* genes.

## Data Availability

Data will be made available on request.

## Supplementary Materials

The following supporting information can be downloaded at: https://www.mdpi.com/article/10.3390/biology14091106/s1. Table S1: Primers used for clone and qRT-PCR of Capsicum annuum; Table S2: The details of the physical and chemical characteristics of CaGRX proteins; Figure S1: Expression patterns of CaGRX genes in response to submergence and hypoxia treatments.
