# Supplementary figures and images for: Systematic Survey and Expression Analysis of the Glutaredoxin Gene Family in Capsicum annuum Under Hypoxia Stress
Source: Biology (Basel). 2025 Aug 22;14(9):1106. doi: 10.3390/biology14091106 (PMC12467025; doi:10.3390/biology14091106)

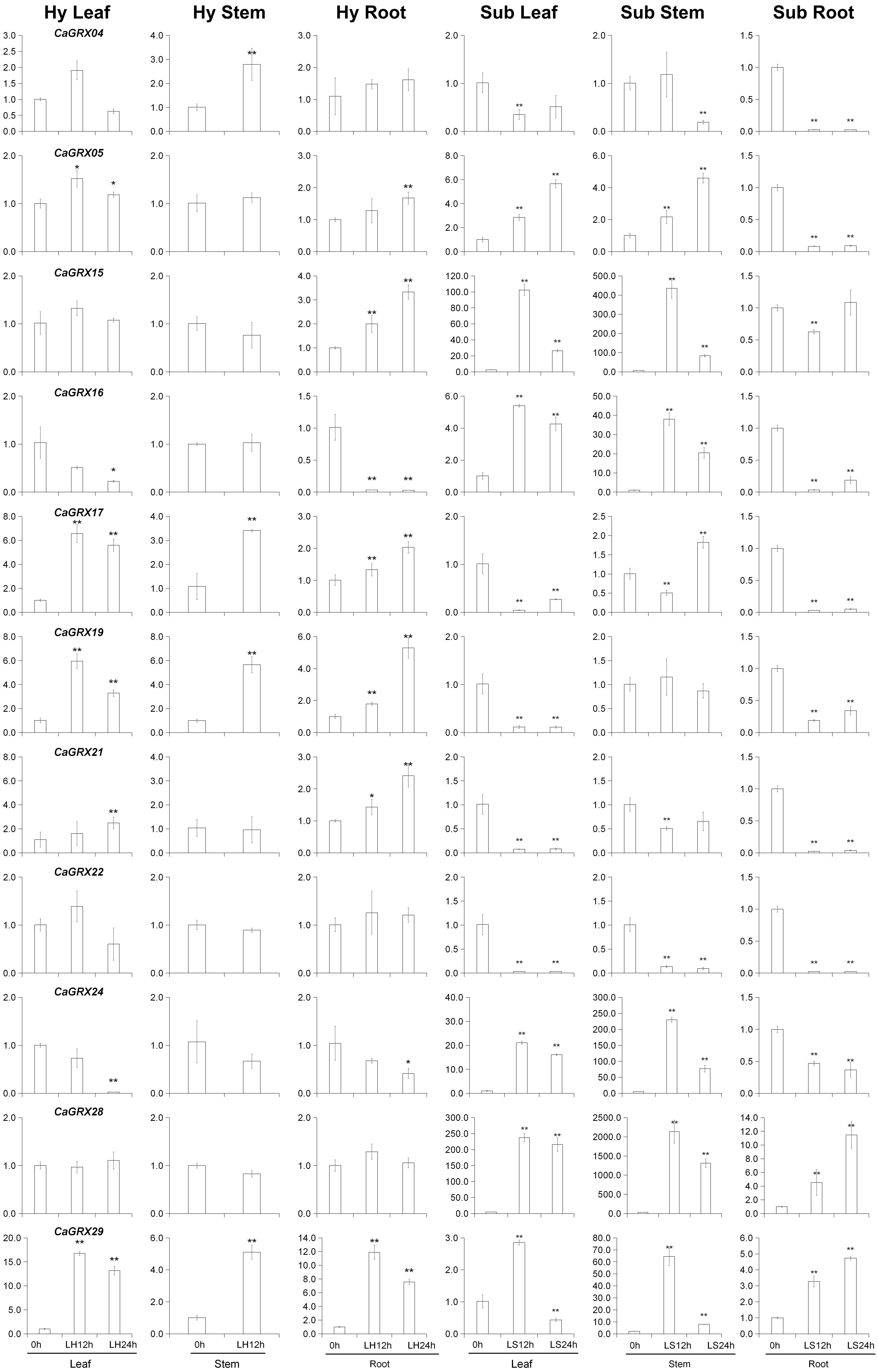

Supplement: Supplementary file 1 [file biology-14-01106-s001.zip › Figure S1.jpg]
